# Short-term results after arthroscopic resection of synovial plicae in the radiohumeral joint: a case series of 64 procedures

**DOI:** 10.1051/sicotj/2017021

**Published:** 2017-06-07

**Authors:** Jens Brahe Pedersen, Pia Kjær Kristensen, Peter Mønsted, Theis Muncholm Thillemann

**Affiliations:** 1 Regional Hospital Horsens Sundvej 30 8700 Horsens Denmark; 2 Department of Orthopedics, Regional Hospital Horsens Sundvej 30 8700 Horsens Denmark; 3 Emergency Department, University Hospital of Aarhus Nørrebrogade 44 8000 Aarhus C Denmark; 4 Department of Orthopedics, University Hospital of Aarhus Tage-Hansens Gade 2 8000 Aarhus C Denmark

**Keywords:** Synovitis, Plica, Elbow, Arthroscopy, Impingement

## Abstract

*Introduction*: Painful Synovial Plicae (SP) in the posterolateral corner of the radiohumeral joint may be confused with lateral epicondylitis. The SP may impinge between the radial head and the humeral capitellum causing pain and snapping. The aim of this study was to evaluate the short-term results after arthroscopic plica resection of the elbow.

*Methods*: In this case series, we included a consecutive series of 64 arthroscopies (60 patients) with arthroscopic plica resection of the elbow. Inclusion criteria were six months of lateral elbow pain and unsuccessful conservative treatment. Patients had either ultrasonography verified plicae or pain on palpation of the plica. Patients were evaluated with an Oxford Elbow Score (OES) preoperatively, after three months and after mean 22 months (range: 12–31) of follow-up. Furthermore, baseline characteristics were recorded including, gender, age, body mass index (BMI), occupation, smoking and cartilage damage.

*Results*: The mean age was 44 years (range: 18–66). In 13 elbows, International Cartilage Repair Society (ICRS) grade 1 lesions were present in association with the plica. Preoperatively the mean OES was 19 (95% CI: 17–20). At three and 22 month follow-up the OES increased to 33 (95% CI: 30–36) and 35 (95% CI: 32–38), respectively (*p* < 0.001). Cartilage injury and gender did not affect the outcome. We reported no complications.

*Discussion*: Arthroscopic plica resection of the elbow indicates an improved OES after three and 22 months. A randomized prospective trial is needed to validate the effect of arthroscopic treatment of synovial elbow plicae.

## Introduction

**Figure 1.**
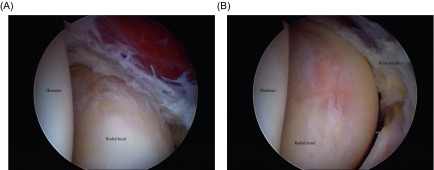
Arthroscopic view of partially resected synovial plica (SP). Notice the osteochondral lesions caused by the SP on the radial head.

*Synovial plica* (SP) in the posterolateral corner of the radiohumeral joint may be confused with Lateral Epicondylitis (LE) [1, 2]. However, the two conditions are very different. Synovial plicae have been described in cadaveric studies and may become inflamed and thus impinge with the radiohumeral joint. The injury has previously been described in throwing athletes and may also be associated with mechanical symptoms, such as snapping or catching [2, 3]. Furthermore, the location of the pain is more posterolateral and not just along the lateral epicondyle or the origin of the extensor tendons [2]. In these cases, the symptoms may be caused by an inflamed SP (Figure 1).

Small case series have previously shown promising results after arthroscopic resection of the SP. However, the cohorts have thus far been small and only few have employed a validated scoring system. Furthermore, the patients in most of these studies were all relatively young throwing athletes [2–4]. The purpose of this study was therefore to evaluate the results after arthroscopic resection of the synovial plicae in the radiohumeral joint in a consecutive series of 64 elbows. Thus, we hypothesized that patients with repetitive strain and symptoms consistent with an inflamed posterolateral elbow plica would benefit from arthroscopic resection.

## Materials and methods

From February 2008 to March 2010, we included 60 patients (64 procedures) who underwent arthroscopic surgical resection of the SP.

All patients had a history of least six months with lateral elbow pain and complaints of mechanical symptoms of catching or snapping. All patients had undergone an unsuccessful nonoperative treatment including supervised physiotherapy, at least one steorid injection and activity modification for at least three months.

Occupation was disclosed by 47 patients (78%), comprised of 22 patients (47%) with jobs regarded as non-manual labour and 25 (53%) defined as manual workers. One patient was unemployed and three patients were on sick leave because of their elbow symptoms.

Patients were selected for arthroscopic plica resection if they met the following inclusion criteria: pain on palpation of the SP, positive ultrasonography (US) and a positive flexion-pronation test (FPT) as described by Antuna and O’Driscoll [4]. The ultrasonography was performed by a single senior orthopaedic consultant in the outpatient clinic. The SP was defined as a triangular shape bordered by hypoechoic rims that exit between the capitellum and the radial head and is isoechoic with the muscles [5]. The FPT was considered positive when a painful snapping was provoked by pronating the forearm while flexing and extending the elbow joint, thus causing the plica to ride over the radial head.

Sixty-seven patients were considered for inclusion in the study. Seven patients were excluded including three patients with confounding comorbidity (rheumatoid arthritis, gout and fibromyalgia), three patients were excluded due to unidentifiable SP during arthroscopy and one patient died of causes unrelated to the elbow surgery before follow-up.

The study group consisted of 17 men (19 procedures) and 43 women (45 procedures) with a mean age of 44 years (95% CI: 42.4–46.4). The mean body mass index (BMI) was 26 kg/m^2^ (95% CI: 24.6–26.6). Twenty-five patients were smokers.

## Patient assessment

Patients were evaluated preoperatively, three months postoperatively and at a mean of 22 months (range 12–31 months) postoperatively.

The primary outcome was a validated version of the Oxford Elbow Score (OES) questionnaire [6, 7]. The OES is a validated scoring system based on 12 questions with a possible score of 0–4 each and thus ranging in total from 0 to 48, a low score denoting greater severity. The OES is subdivided into three main domains, each covered by four questions. The domains are pain, elbow function and social-psychological, each with a possible maximum subscore of 16 points. The total- and subscores were further converted to a metric score allowing easier comparison and interpretation [6]. The range of motion of the elbow joint was evaluated with goniometry.

In total, 60 patients (64 elbows) were included in the study. Fifty-four patients (56 procedures) attended the three month follow-up, thus 6% of procedures (4/64) were lost at the three month follow-up. At the final follow-up, 55 patients (58 procedures) completed the OES questionnaire by mail.

A final OES was completed by mail after mean 22 (range 12–31) months of follow-up.

## Operative technique

All procedures were performed at a single institution by one of the senior authors. A Stryker 45° optic was used. The procedure was performed with the patient in lateral decubitus position. An anterolateral portal was established for inspection of the anterior joint compartment while an anteromedial portal was made with an inside-out technique for instrumentation and resection of the plica. This resection was performed with a 4.5 mm shaver and/or an Artrocare^®^ radio frequency ablation device. A posterolateral portal was then established in order to inspect the posterior and lateral compartments and allow complete resection of the plica. A portal for instrumentation was established in the soft spot between the radial head, the lateral epicondyle and the olecranon. Cartilage damage was graded according to the International Cartilage Repair Society Score (ICRS) which was noted by the surgeon in the operation report [8].

All surgeries were performed as outpatient surgeries and all patients were discharged with a standard rehabilitation programme beginning the first postoperative day. This regimen consisted of active exercises without resistance for two weeks with a gradual increase in resistance the following two weeks. The training was unsupervised.

## Statistical analysis

Statistical analysis was performed with STATA, version 12.0 (StataCorp LP, College Station, TX, USA). Normality of continuous data was inspected by frequency (Q-Q plots). Descriptive analyses were performed to describe baseline characteristics and outcome measures. Continuous data was reported as means and standard deviation (parametric) or as medians and percentiles and categorical data as numbers with percentages. Oxford Elbow Scores were compared between the follow-up times by use of a paired Student’s *t*-test. All tests were performed with a 95% confidence interval. The level of significance was set at *p* < 0.05.

## Results

Preoperatively, the mean OES was 19.0 (95% CI: 17.3–20.3) and increased significantly to a mean OES 33.5 (95% CI: 30.2–36.0) at the three month follow-up (*p* < 0.001). The OES did not increase significantly further at the 22 month follow-up: OES = 35.6 (95% CI 32.4–38.8; *p* < 0.08; Table 1).

**Table 1.** Oxford Elbow Scores and subscores before and after arthroscopic resection of the posterolateral synovial plica of the elbow.

	Preoperative *n* = 64 (95% CI)	Three month follow-up *n* = 55 (95% CI)	22 month follow-up *n* = 57 (95% CI)	*p*-Value (preoperative OES ≠ OES at three months)
OES, total*	19.0 (17.3–20.3)	33.5 (30.2–36.0)	35.6 (32.4–38.8)	*p* ≤ 0.0001
%	39.5	69.7	74.1	N.a.
Pain domain**	5.3 (4.7–5.9)	9.8 (8.7–11.1)	11.3 (10.2–12.3)	*p* ≤ 0.0001
%	33.1	61.25	70.6	N.a.
Psychosocial domain	3.8 (3.2–4.4)	9.4 (8.2–10.5)	10.8 (9.6–11.9)	*p* ≤ 0.0001
%	23.8	58.8	67.5	N.a.
Elbow function	8.5 (7.5–9.4)	12.9 (11.9–13.9)	13.6 (12.8–14.4)	*p* ≤ 0.0001
%	53.1	80.6	85.0	N.a.

* Maximum possible OES total score = 48;

** Maximum possible OES subscore = 16;

CI = confidence interval; N.a. = not applicable.

The OES pain domain was preoperatively 5.3 (95% CI: 4.7–5.9) and increased to 9.8 (95% CI: 8.7–11.1) and 11.3 (95% CI: 10.2–12.3) after three month and 22 month follow-up, respectively (Table 1).

The subscore for elbow function was preoperatively 8.5 (95% CI: 7.5–9.4), 12.9 (95% CI: 11.9–13.9) at three months and 13.6 (95% CI: 12.8–14.4) at final follow-up.

The sociopsychological subscore was preoperatively 8.0 (95% CI: 3.2–4.4), at three months 12.9 (95% CI: 11.9–13.9) and at final follow-up 13.6 (95% CI: 12.8–14.4).

At final follow-up, 26 patients returned 27 (47%) OES questionnaires with a score ≥ 40, which we defined as a satisfactory result.

In 13 elbows (20%), ICRS grade 1 lesions consisting of small fissures, indentations or cracks were present on the radial head in association with the plica. When data was stratified according to cartilage injury and gender, the outcome was not affected.

Seven patients (12%) had a decreased range of motion in their elbow preoperatively. One patient, who had an extension deficiency of 30° preoperatively, improved to a 20° deficiency in three months. At final follow-up, all patients had normal range of motion, except for one patient who had a flexion deficit of 20° due to pain. Patients were assessed for potential complications including infection, complex regional pain syndrome and nerve injuries. We reported no complications to the surgeries.

## Discussion

Synovial plicae are remnants of the embryonic development of the joint [9, 10]. It has been hypothesized that the synovial fold may hold a function similar to the meniscus of the knee and even aid in proprioception [4]. It is believed that these plicae can become hypertrophic from repetitive strain and trauma [2, 3]. This hypertrophy may then cause the plica to impinge between the radial head and the humeral capitellum causing pain and snapping. Kim et al. [2] reported arthroscopic results on 12 throwing athletes and attributed the plicae to this repetitive strain and trauma. Both Antuna and O’Driscoll [4] and Rhyou and Kim [11] described the pain caused by SP as a plausible differential diagnosis to lateral epicondylitis.

In their 12 patient series, Kim et al. reported 75% of their patients to have an excellent outcome rated by Modified Elbow Scoring Scale (MESS), 17% to have a good outcome and 1 patient had a fair outcome following plica resection. Furthermore, 92% were able to return to sports [2]. None of these patients suffered from lateral epicondylitis.

Ruch et al. [12] reported on 10 patients demographically more similar to our patients using the Disability of the Arm, Shoulder and Hand (DASH) score for evaluation but this score was only performed once postoperatively at mean 25 month follow-up and four patients had more than one operation performed on the elbow. All patients had a SP which was debrided. Two patients (20%) had a DASH score of 33 and 37. Babaqi et al. [13] treated 31 patients for 33 cases of lateral epicondylitis with arthroscopic debridement. In this series, debridement of the undersurface of extensor carpi radialis brevis as well as resection of any impinging SP was performed. Patients improved significantly on DASH (from 24.46 to 4.81) and Visual Analogue Scale (from 8.64 to 1.48) at a mean follow-up of six months. Of the 31 patients, 93.5% reported a satisfactory outcome.

In our case series, 27 elbows (47%) could be defined as having satisfactory joint function (OES ≥ 40) at final follow-up. Twelve patients (19%) had an OES of 46 or greater corresponding to what Guyver et al. found to be the normal value for a healthy population [14]. Thus, our results are in contrast to those of Kim et al. which might be explained by the relatively young and healthy athletes included in their study (mean 22 years) whereas our population was non-athletic and had a mean age of 44 years.

The subscores of our study indicate that functional outcome at final follow-up can be interpreted as satisfactory. However, the outcome of the sociopsychological- and pain domains influences the OES negatively.

Selecting patients eligible for arthroscopic debridement remains a challenge since preoperative imaging is difficult: Cerezal et al. reported a significant overlap in thickness of symptomatic and asymptomatic plicae when reviewing magnetic resonance imaging (MRI) scans [10]. However, MRI can detect secondary signs of elbow synovial fold syndrome, such as posterolateral synovitis and chondromalacia, in either the anterolateral part of the radial head or on the capitellum [9, 10]. Ultrasonography has a high sensitivity and specificity and can also determine inflammation by Doppler but still has limitations regarding the lateral aspects of elbow plicae [5]. Although preoperative diagnostics such as ultrasonography and MRI have limitations, they can aid in the selection of patients eligible for arthroscopy by identifying not only plicae, but also concurrent pathology [5, 9, 10]. Relying on the FPT and US alone may not provide enough diagnostic information before choosing surgical treatment of SP, which could partially explain the limited satisfaction of our patients.

## Limitations

The main limitation of this study is that although the surgical cases were recruited over a sustained period, our sample only represented one surgical centre. The elbow conditions undergoing surgical treatment may therefore not be typical of other centres which decreases the external validity.

This study did not have a control group to compare the long-term effects of non-surgical treatment of elbow plicae.

A combined MRI- and ultrasonography scan in all patients would have been helpful in determining if some patients had more than one pathology in the elbow which could explain why so many patients did not get a better outcome.

## Conclusion

Arthroscopic plica resection in the elbow improved OES after three and 22 months. Forty-seven percent of all patients reported an OES result of ≥ 40 indicating satisfactory joint function at 22 months. Only seven patients (12%) reported complete remission of symptoms at final follow-up and therefore we do not recommend plica resection without thorough clinical and imaging diagnostics to rule out coinciding morbidity. A randomized prospective trial is needed to validate the effect of arthroscopic treatment of synovial elbow plicae.

## Conflict of interest

JBP, PKK, PM and TMT certify that they have no financial conflict of interest (e.g., consultancies, stock ownership, equity interest, patent/licensing arrangements, etc.) in connection with this article.
